# Tea plantations and their importance as host plants and hot spots for epiphytic cryptogams

**DOI:** 10.1038/s41598-021-97315-2

**Published:** 2021-09-14

**Authors:** Grzegorz J. Wolski, Renata Piwowarczyk, Vítězslav Plášek, Martin Kukwa, Karolina Ruraż

**Affiliations:** 1 Department of Geobotany and Plant Ecology, Faculty of Biology and Environmental Protection, University of Lodz, Banacha 12/16, 90-237 Lodz, Poland; 2 Center for Research and Conservation of Biodiveristy, Department of Environmental Biology, Institute of Biology, Jan Kochanowski University, Uniwersytecka 7, 25-406 Kielce, Poland; 3 Department of Biology and Ecology, University of Ostrava, Chittussiho 10, 710 00 Ostrava, Czech Republic; 4 Department of Plant Taxonomy and Nature Conservation, Faculty of Biology, University of Gdańsk, Wita Stwosza 59, 80-308 Gdańsk, Poland

**Keywords:** Plant ecology, Plant sciences, Environmental sciences, Environmental impact, Ecology, Biogeography

## Abstract

Bryophytes and lichens are outstanding bioindicators, not only of the plant community in which they develop, but also the substrates on which they grow. Some epiphytic cryptogams, particularly the rare ones, are stenotopic and require a long habitat continuity, for example substrates such as old trees. It could also be a tea plantation, this is because the shrubs are not felled, and most of them may have several dozen years. In addition, the shrubs are not subject to sudden changes in microclimatic conditions as only the young leaves are harvested. As the importance of tea plantations as host plants for mosses and lichens has not yet been studied, the present study examines the species diversity of cryptogams of two tea plantations in Georgia (Caucasus). The study also examines the phytogeography, spatial pattern, environmental conditions and ecological indicators of the cryptogams. Thirty-nine cryptogam taxa were identified; typical forest taxa dominated, even in the absence of typical forest communities. Some of these species are obligatory epiphytes, rare or even critically endangered in most European countries (e.g., *Orthotrichum stellatum*, *O*. *stramineum*, *Lewinskya striata*). The fairly abundant record of such species on tea plantations indicates the importance of these phytocoenoses for the preservation of rare species, and indicates that these habitats are hot spots for these cryptogams in otherwise changed envirnonment. Additionally, as indicated the analysis of the species composition of individual plantations and the mathematical analysis made on this basis, plantations differ from each other. Another interesting result is also the spatial distributions of cryptogams on tea bushes resemble those of forest communities and lichens seems to be more sensitive than bryophytes to antropogenic changes of environment.

## Introduction

As well as being used to make the oldest and most popular drink in the world, the leaves of the tea plant, *Camellia sinensis* (L.) Kuntze (Theaceae), are important components in medicine and pharmacology^1^. Tea plantations are cultivated all over the world on almost all continents, in 58 countries. However, they today are mainly grown in Asia, Africa, South America, and around the Black and Caspian Seas, which is related to specific climate and habitat requirements. Currently more than 75% of the world's tea production comes from: China, India, Sri Lanka, Kenya and Vietnam, while, the total land under tea cultivation was 3.36 million hectares and production was 4.78 million tonnes^2,3^.Tea plantations have also been established in Georgia (Caucasus region), as due to its proximity to the Black Sea. Western Georgia has a humid and subtropical climate which is favourable for tea cultivation. Tea in Georgia is grown in four regions, viz*.* Adjara, Guria, Samegrelo and Imereti^4^, where it has been cropped since the mid-nineteenth century. It should be emphasized that in the 1960–1970s Georgia was the main tea producer in the Soviet Union, but after 1991, the tea sector in Georgia collapsed. Now, the Georgian government and various agencies are currently trying to reactivate many plantations for use also as tourist attractions^4^.

Epiphytic bryophytes and lichens (lichenized fungi) form an integral component of almost all land ecosystems, including forests and shrub vegetation, and are an important and irreplaceable component of species diversity^5,6^. Moreover, both groups of organisms have important ecosystem functions as they increase structural complexity, influence nutrient cycles and moisture retention, and provide habitats, food and nest material for animals^7–9^. Additionally, due to the strong relationship between bryophytes and lichens, with the overgrown substrate and the plant community in which they are recorded these organisms are used in phytosociological studies^10–15^.

In forests, epiphytic bryophytes and lichens grow under more demanding climate-based constraints than terrestrial plants^16^. Their dependence on the atmospheric supply of both water and nutrients make them good indicators for habitat characterization^17–21^. With increasing tree height, the vertical distribution of epiphyte communities is influenced by decreasing humidity and increasing light intensity, wind and evaporation. Hence, many epiphytic bryophytes and lichens prefer shaded places^22^. They may suffer from high light intensity and water deficits when living on the bark of large trees, thus affecting their growth and physiological attributes: they have low light-saturated photosynthetic rates, low dark respiration rates and light saturation points when compared with phanerogams^23^. In addition, some epiphytic bryophytes and lichens, particularly the rare ones, are stenotopic and require long habitat continuity, for example, substrates such as old or large trees^17,24^.

Lichens and bryophytes are an important component in several open and exposed ecosystems, such as drylands, sand dunes and roadside trees. As a component of biocrusts they may play an important role in the restoration of drylands and influence edaphic factors in biocrust establishment and development. They also contribute to the biodiversity in non-forest ecosystem (e.g., roadside trees, sand grasslands) as numerous species can inhabit such environments^25–32^.

Epiphytic cryptogams have not been studied with the same intensity in all types of habitats in the world. Their species diversity was monitored in different land-use types in tropical areas in detail, e.g., America and Indonesia^33^, and changes in species richness from the natural forest throught the modified habitats till exposed ecosystems varied greatly, from 10% species loss in secondary forest to 65–80% in extensively agriculturally used habitats. Similarly, on isolated trees and shrubs in Ecuadorian pastures, only 30–35% fewer species than in the adjacent primary forest were recorded than in forest areas. Nevertheless, trees and shrubs in open ecosystem are rich in lichens and other groups of epiphytes, and may play an important role in biodiversity conservation in areas where the forest has been revise by human management^34–36^. As it can be expected, shade-dwelling epiphytes were often replaced by sun-demanding species in the drier land-used types of habitats^33^. High diversity of the epiphytic bryophytes can be found also in natural open habitats with shrubs vegetation, such as fynbos in South Africa or chaparral in South America, which offer suitable conditions for epiphytic mosses. A recently described new species, *Orthotrichum karoo* F. Lara, Garilleti & Mazimpaka, is an interesting case^37^ as it seems not to be an accidental occurrence of the species in such ecosystems, but rather example of the speciation linked with this open, xerophytic habitat. The species represents unique features of both, the gametophyte and the sporophyte, which could be interpreted as adaptations to this type of ecosystem. The same peristome constitution has been described for two other species, the Mediterranean *O. acuminatum* H. Philib.^38^ and the Californian *O. anodon* F. Lara, Garilleti & Mazimpaka^39^. Not only forest but also open ecosystems should be considered as important habitats for the existence and development of epiphytic populations and could be given attention in terms of conservation.

In managed forests, populations of bryophytes and lichens have decreased in size or even become extinct because of the effects of silvicultural measures^22,24,40^. However, many species of bryophytes and lichens in shrub vegetation are flourishing, and in this regard, tea plantations can act as substitute habitats. The presence of cryptogams is favoured by the fact that the woody plants are not felled and hence, no sudden changes occur in microclimatic conditions. The density of vegetation and the canopy formed by the shrubs also help to create favourable humidity and at the same time non-aggressive light conditions for the development of mossy and lichen vegetation. In addition, the growth of shrub stems may increase species diversity and the dynamism of their communities due to changes in microclimatic factors such as moisture, light, and bark characteristics, as well as greater competition among epiphyte individuals^22^.

In recent years, several studies have analysed the effects of forest management on the species diversity and composition of epiphytic bryophytes and lichens in coniferous and deciduous forests in North America, Europe and Asia^6,41–46^. In addition, few papers have focused on the occurrence of epiphytes in shrub vegetation, or on cryptogams in tea plantations, and existing research is usually fragmentary^47,48^. However, tea plantations and their importance as host plants for epiphytic mosses and lichens have not yet been studied.

The aim of this study focuses on three main points: evaluating lichen and bryophyte diversity in two Georgian tea plantations; analyzing the vertical distribution patterns of lichens and bryophytes on tea shrubs, and assessing the influence of environmental factors on the distribution of the analyzed cryptogams.

## Materials and methods

### Study area and data collections

**Figure 1:**
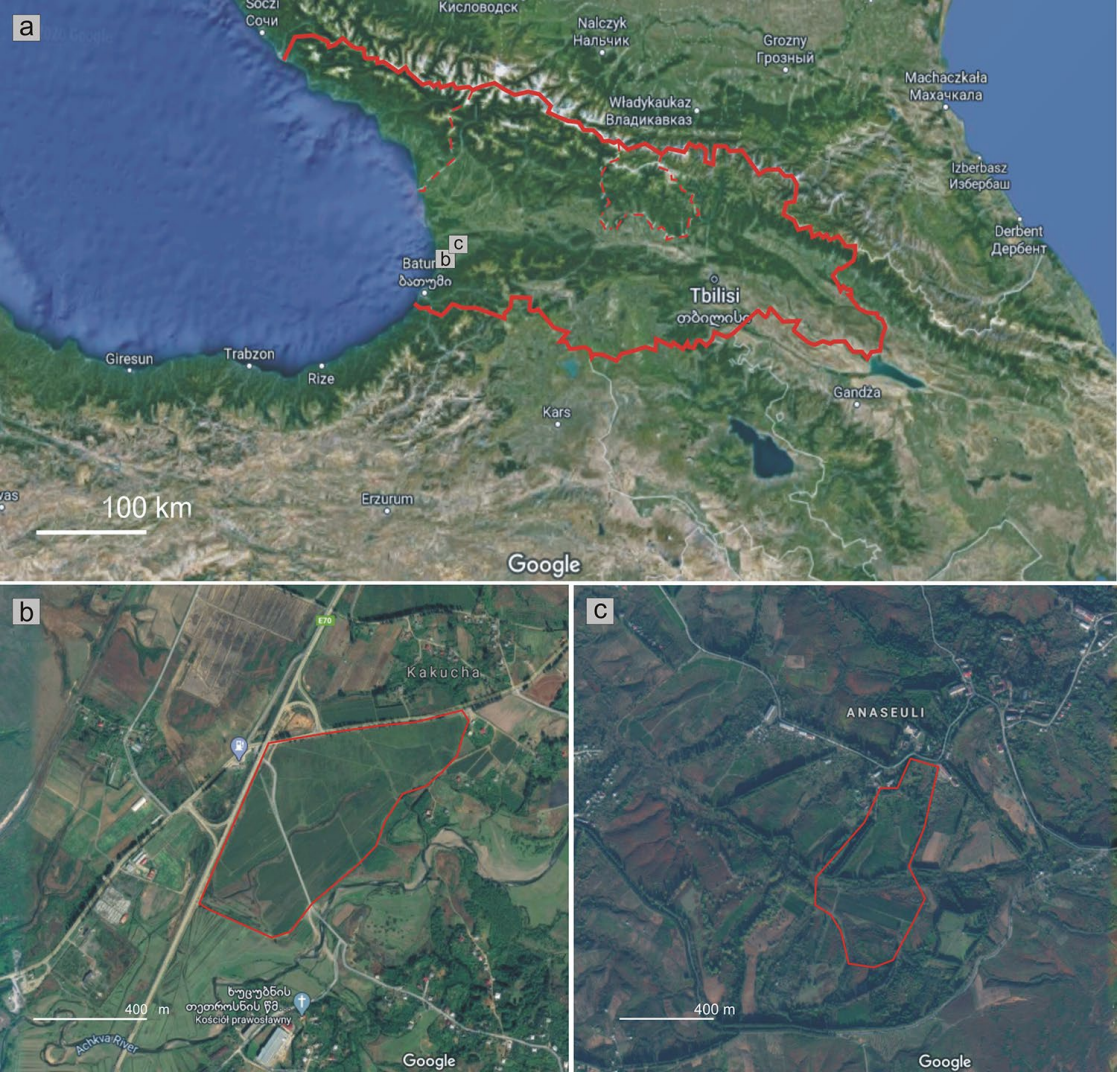
The location of the studied areas with regard to the borders of Georgia (**a**) and those of the studied tea plantations in Kobuleti (**b**) and Ozurgeti (**c**). Red dashed lines in (**a**) refer to Abkhazia and South Ossetia. Map was created on the basis of Google maps (https://www.google.pl/maps/place/Gruzja) by G. J. Wolski in the CorelDRAW 12.0 Graphic program.

The study was conducted in July of 2014, 2017 and 2018 on two tea plantations in western Georgia (Caucasus) (Fig. 1a): Kobuleti (Fig. 1b) and Ozurgeti (Fig. 1c).

In order to describe the climatic conditions, the monthly averages of weather data for each locality was determined based on monthly minimum and maximum temperatures (°C) and precipitation (mm) (Supplementary Table S1).

**Figure 2:**
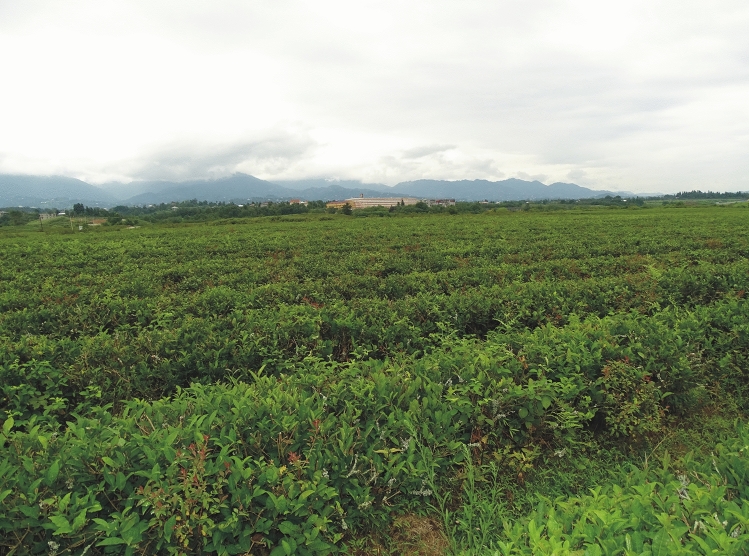
Tea plantation near Kobuleti (photo by R. Piwowarczyk, 29 July 2018).

The Kobuleti plantation is located SW of Kakucha and N of Khutsubani village, near Kobuleti in the Adjara region: 41°49′ 43.28″ N, 41°49′ 20.21″ E, (10–)15(–20) m elevation. The plantation occupies an area of approximately 0.5 km^2^ (Fig. 1), and is located 4 km east of the Black Sea, in a lowland area surrounded by arable fields and the Kobuleti Bypass. The Achkva River and its tributaries flow through the central and southern parts of the plantation (Figs. 1b and 2). The plantation is heavily overgrown with young tea shoots; however, the lowest layer is composed of the invasive grass *Microstegium japonicum* (Miq.) Koidz., with an admixture of *Pteridium tauricum* (C. Presl) V.I. Krecz. ex Grossh. and *Hypolepis punctata* (Thunb.) Mett. ex Kuhn, as well as *Spiraea japonica* L. f. and *Rubus* sp. div., while species of *Juncus* occur in more humid places. The average annual minimum temperature in the Kobuleti plantation for the analysed period ranged from 10.2 to 11.2 °C; the highest annual minimum temperatures was recorded in 2014 and the lowest in 2017. The average annual maximum temperature in the region ranged from 19.1 (in 2013) to 20.2 °C (in 2014), average annual precipitation ranged from 163.9 mm (in 2017) to 221.8 mm (in 2016)^49^.

**Figure 3:**
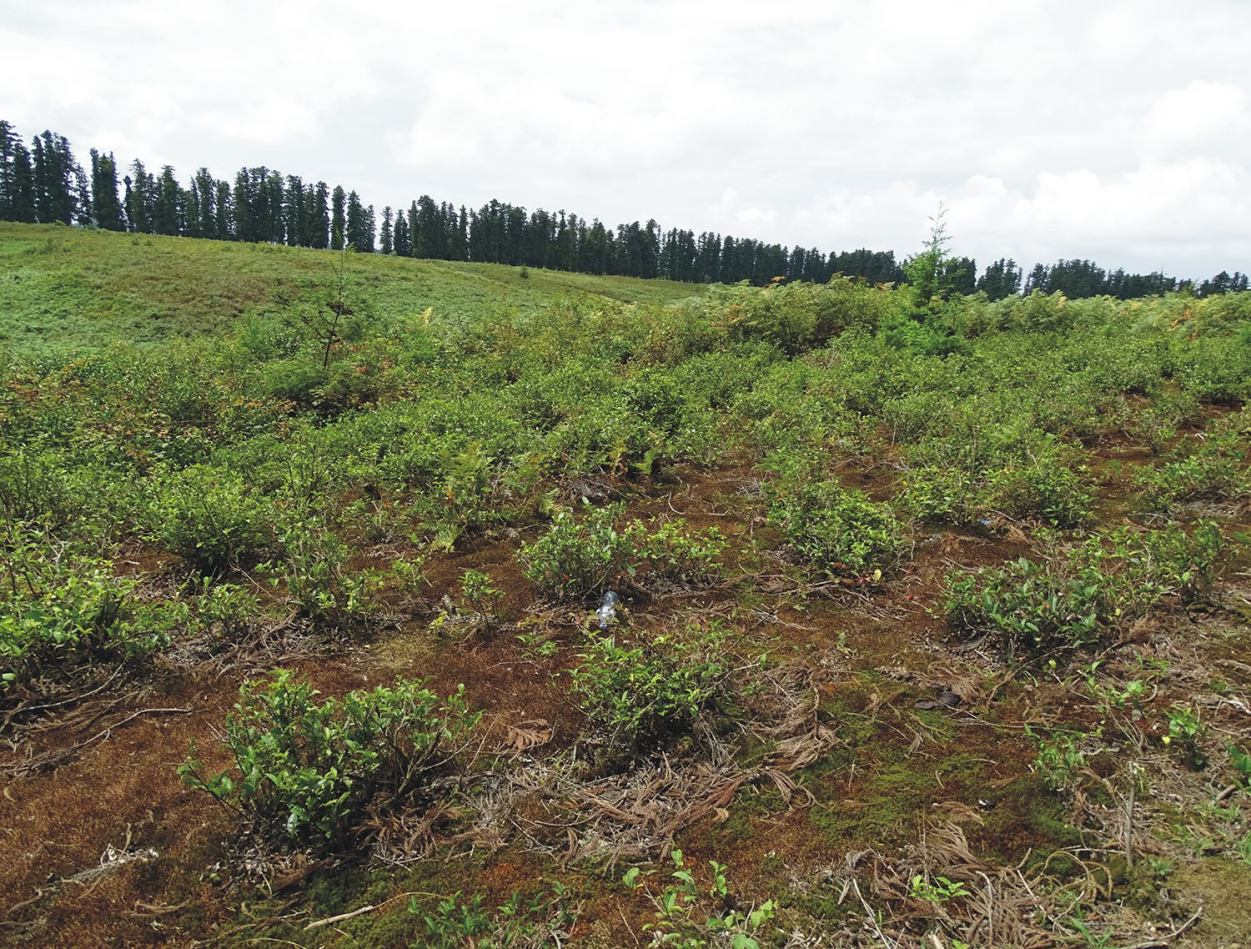
Uniform turfs formed by *Polytrichum* on the soil around tea bushes on the plantation in Ozurgeti (photo by R. Piwowarczyk, 29 July 2018).

The Ozurgeti plantation is located SW of Anaseuli near Ozurgeti, in the Guria region: 41°54′13.81″ N, 41°58′38.56″ E, (115–)120(–140) m elevation. The plantation occupies an area of approximately 0.3 km^2^ (Fig. 1), and is situated ca 20 km east of the Black Sea, on a slightly hilly terrain, in the vicinity of arable fields. Many local roads are lined with stately and tall *Cryptomeria japonica* (Thunb. ex L. f.) D. Don trees, some of which are found inside the plantation area. Compared to the Kobuleti plantation, this one is much more cropped, with more solar radiation reaching the interior (Figs. 1c and 3) and occupies a much drier habitat. Also, unlike Kobuleti, the lowest layer is composed of mosses rather than vascular plants. Amongst the weeds, numerous *Hypolepis punctata*, *Pteridium tauricum* and *Spiraea japonica* dominate, with a slight occurrence of *Microstegium japonicum*. The plantation appears more neglected, and the soil is often covered with a dense mossy layer. Established in 1935 by the Anaseuli Tea Factory, it is one of the oldest tea production facilities in Georgia. The average annual minimum temperature in the Ozurgeti ranged from 10.1 (in 2017) to 11.0 °C (in 2014) and the maximum temperature from 19.1 (in 2013) to 20.1 °C (in 2014). The average annual precipitation in the region varied from 155.9 (in 2017) to 209.2 mm (in 2016)^49^.

### Sampling design and environmental predictors

Detailed floristic-ecological documentation (understood as a list of taxa growing on certain habitats and substrates) was taken on each plantation in homogeneous phytocenosis at randomly-selected positions. All plantations were evenly, thoroughly penetrated. In their area, the number of test sites was dependent on the area of each of them. Wherein 38 of them were made for Kobuleti, and 21 in Ozurgeti; at each position, the occurrence of all lichens and bryophytes found in each tea shrub (and their 20 cm surrounding area) was recorded (added as Supplementary Tables S2–S3), also including the specific substrate (epigeic, epixylic, epiphytic habitat) in which they appear and grow. Photographic documentation was made for each positions. The collected material was determined to species level; however, *Fellhanera* sp., were only identified to the genus as only thalli without apothecia were found and no detailed determination was possible (Table 1).

Based on data retrieved from both plantations relating to the occurrence of the each species the general frequency of the all recorded taxa was defined (Table 1). Wherein, it was determined on a three-level scale: 1–30% of the test sites—rare species; 31–66%—frequent species; more than 66% of the test sites—common species (Table 1).

**Figure 4:**
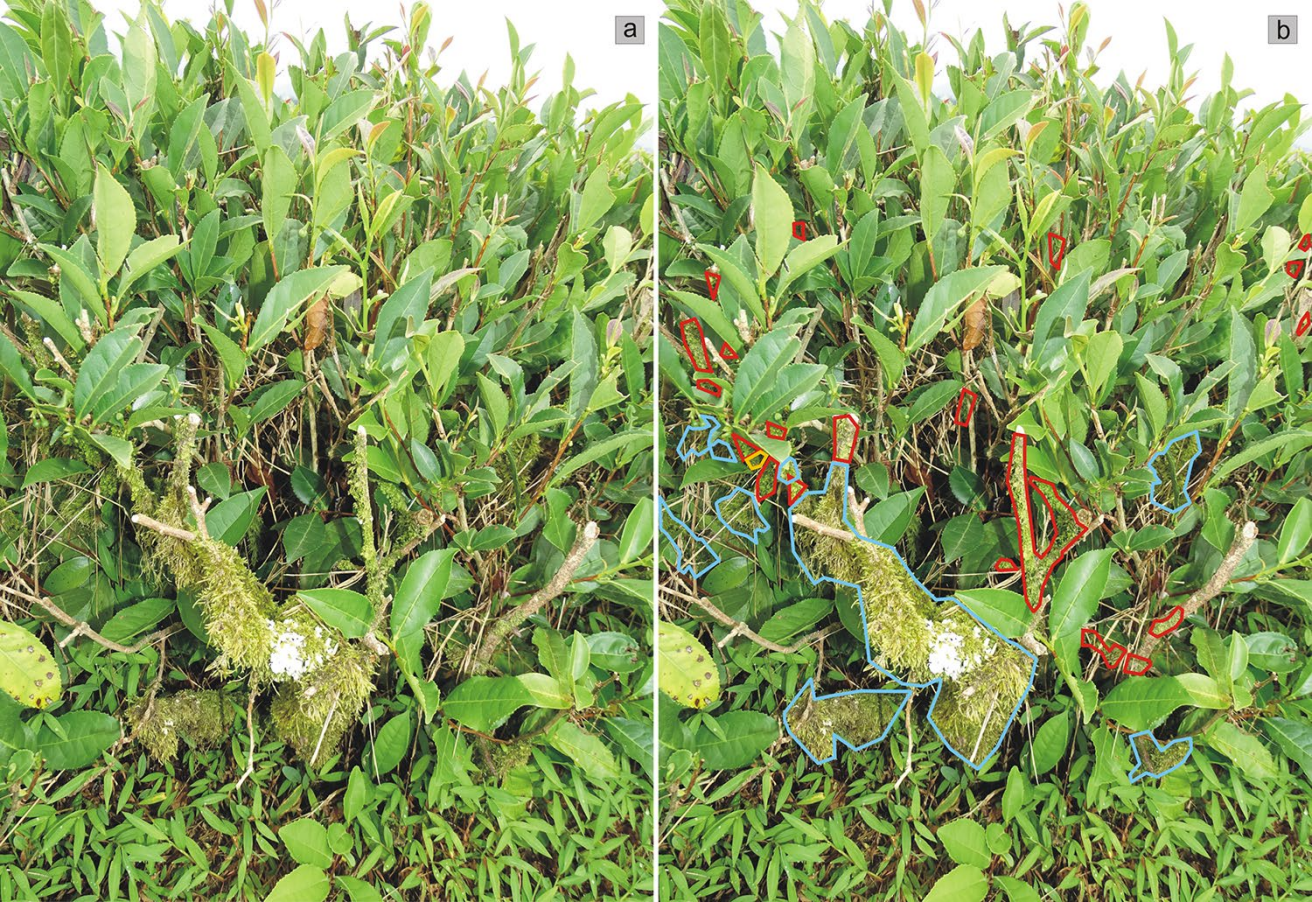
Example distribution patterns and coverage by selected taxa—Kobuleti plantation. (**a**) Output photo, (**b**) photo with mosses taxa marked (photo by R. Piwowarczyk, 29 July 2018). Explanation: *Hypnum cupressiforme*—blue, *Orthotrichum* sp. div.—orange, *Radula complanata*—red.

Additionally, the area they covered was specified according to a fourfold classification: the lower, middle or upper part of the tea shrub, or on the surrounding soil (Table 1). This classification was referred to the height at which individual taxa were recorded on the tea bush. From the ground surface to 40 cm it was the lower zone; 41–80 cm the middle, above 80 cm the upper zone. Having the above data collected on this basis, the presence of individual taxa for each zone was determined. Thus, the spatial distribution of individual taxa was determined (Fig. 4), with examples being given in images from individual plantations (Figs. 6 and 7, Table 1).

The meteorological datasets were obtained for 2013–2017 and 2018 (from January to July) records. The weather data was extracted from the ‘WorldClim’ database^49^ and is available for download from http://www.worldclim.org. Climatic information of the two localities used in the analysis is provided in the Supplementary Table S1.

Ecological indicator values were assigned to taxa. These were adopted for lichens after Wirth^50^, and for bryophytes after Ellenberg et al.^51^, with exception of species that did not have index numbers and *Fellhanera* sp. On this basis two indicator values were analysed: F—humidity and L—insolation. In addition, all analysed taxa were given a six-letter code derived from the first three letters of the genus and species name (Supplementary Tables S2 and S3).

Samples of the identified bryophytes and lichens growing on the tea bushes and the surrounding soil were collected. However, this does not apply to legally protected, rare and endangered plants, other specimens were deposited in the following herbaria: Jan Kochanowski University in Kielce (KTC), University of Gdańsk (UGDA) and University of Lodz (LOD). Field studies, including the collection of plant material was compiled with relevant institutional, national, and international guidelines and legislation, also permissions were obtained for the collection of plants and plant materials from the plantations. The lichens were named after Smith et al.^52^, and the mosses after Hodgetts et al.^53^. G. J. Wolski and V. Plášek were responsible for the identification of bryophytes and M. Kukwa for lichens.

### Statistical analysis

The PAST v. 4.06b statistical package was used for the calculations. Species richness was determined by specifying numbers of species found on both plantations. The Jaccard (d) measure was taken as the measure of similarity. The permutational multivariate analysis of variance (PERMANOVA) was used to determine the significance of statistical differences in the occurrence of species on individual plantations and tea shrubs zones. The relationships between species and their places of occurrence were determined using principal components analysis (PCA). The H Shannon index was adopted as a measure of species diversity, while, the t-test was used to determine the differences between the differentiation indices.

## Results

### Species composition and habitats

During the study, 39 taxa of cryptogams were recorded: 30 bryophytes (four liverworts and 26 moss taxa) and only nine lichens. Among the listed species, the genera *Hypnum* Hedw. and *Lewinskya* F. Lara, Garilleti & Goffinet dominated (both represented by three species), fewer *Frullania* Raddi and *Polytrichum* Hedw. (by two species). The remaining genera were represented by one species (Table 1).

**Table 1:** Species recorded on individual plantations.

No	Species	Ozurgeti	Kobuleti	Habitat	Zone	Frequency	Threatened
**Liverworts**							
1	*Frullania dilatata* (L.) Dumort	+	+	F	M, U	Fr	
2	*F*. *tamarisci* (L.) Dumort		+	F	M, U	Fr	+
3	*Metzgeria furcata* (L.) Corda		+	F	M, U	Fr	
4	*Radula complanata* (L.) Dumort	+	+	F	U	C	
**Mosses**							
5	*Alleniella complanata* (Hedw.) S.Olsson, Enroth & D.Quandt	+	+	F	M	Fr	
6	*Atrichum undulatum* (Hedw.) P.Beauv	+	+	G	S	Fr	
7	*Ceratodon purpureus* (Hedw.) Brid	+		G	S	R	
8	*Eurhynchium striatum* (Hedw.) Schimp		+	G	S	R	
9	*Exsertotheca crispa* (Hedw.) S.Olsson, Enroth & D.Quandt	+	+	F	M	Fr	
10	*Hypnum andoi* A.J.E.Sm	+	+	K, F	S, M	Fr	+
11	*H*. *cupressiforme* Hedw	+	+	K, F	S, L, M	C	
12	*H*. *cupressiforme* var*. filiforme* Brid	+	+	K, F	S, L, M	C	
13	*Homalothecium lutescens* (Hedw.) H.Rob	+		F	M	Fr	
14	*Isothecium alopecuroides* (Lam. ex Dubois) Isov		+	F	M	Fr	
15	*Jochenia pallescens* (Hedw.) Hedenäs, Schlesak & D.Quandt	+		K, F	S, M	Fr	+
16	*Kindbergia praelonga* (Hedw.) Ochyra	+	+	G	S	R	
17	*Lewinskya affinis* (Brid.) F.Lara, Garilleti & Goffinet		+	F	M, U	R	+
18	*L*. *speciosa* (Nees) F.Lara, Garilleti & Goffinet		+	F	M, U	R	+
19	*L*. *striata* (Hedw.) F.Lara, Garilleti & Goffinet	+	+	F	M, U	R	+
20	*Leucodon sciuroides* (Hedw.) Schwägr		+	F	M, U	R	
21	*Neckera pumila* Hedw		+	F	M, U	R	
22	*Orthotrichum stellatum* Brid	+	+	F	M, U	R	+
23	*O*. *stramineum* Hornsch. ex Brid		+	F	M, U	R	+
24	*Polytrichum commune* Hedw		+	G	S	Fr	
25	*P*. *longisetum* Sw. ex Brid	+	+	G	S	R	
26	*Plagiomnium affine* (Blandow ex Funck) T.J.Kop	+	+	G	S	Fr	
27	*Platygyrium repens* (Brid.) Schimp		+	F	M	Fr	
28	*Stereodon callichrous* (Brid.) Lindb	+	+	K, F, G	S, L, M	C	+
29	*Thuidium delicatulum* (Hedw.) Schimp	+	+	G	S	R	
30	*Ulota crispa* (Hedw.) Brid	+	+	F	M, U	Fr	+
**Lichens**							
31	*Amandinea punctata* (Hoffm.) Coppins & Scheid		+	F, K	S, M, U	R	
32	*Bacidia laurocerasi* (Delise ex Duby) Ozenda & Clauzade	+	+	F	U	Fr	
33	*Cladonia rei* Schaer		+	G	S, M, U	R	
35	*Evernia prunastri* (L.) Ach	+	+	F, K	S, M, U	R	
36	*Fellhanera* sp. (only pycnidia)	+	+	F	U	Fr	
37	*Flavoparmelia caperata* (L.) Hale	+	+	F	M, U	Fr	
38	*Parmotrema perlatum* (Huds.) M. Choisy	+	+	F	M, U	Fr	
39	*Punctelia subrudecta* (Nyl.) Krog	+		F	M, U	R	
40	*Ramalina farinacea* (L.) Ach		+	F	M, U	R	
		**25**	**35**				**10**

Explanation: habitat (G—epigeic, K—epixylic, F—epiphytic); four zones on and around the tea bushes (L—lower, M—middle, U—upper zone, and S—soil); frequency of individual species (R—rare, Fr—frequent and C—common species); threatened species selected on the basis of literature cited throughout the article.

Thirty-five taxa were recorded on the Kobuleti plantation (87% of all recorded on both plantations), including 14 exclusive species (e.g., *Frullania tamarisci*, *Metzgeria furcata*, *Ramalina farinacea*), while 25 species were recorded on the Ozurgeti plantation (61% of all species), including only three exclusive species: *Ceratodon purpureus*, *Jochenia pallescens* and *Punctelia subrudecta* (Table 1).

Jaccard's measure (d = 0.4872) calculated for both plantations showed no remarkable similarity between the studied areas. Both plantations contain 49% of the common taxa (21 taxa), wherein only two of them being the most common on almost every shrub—*H*. *cupressiforme* and *H*. *cupressiforme* var. *filiforme*. On the other hand, among 39 of all listed species, as much as 46% are taxa exclusive to the surveyed plantations. In Ozurgeti, the most exclusive species are photophilous and require less moisture, e.g.: *Ceratodon purpureus* (L 8; F 2), *Punctelia subrudecta* (L 7; F 3). While, among the exclusive species of Kobuleti plantations, taxa with higher humidity and lower requirements to light conditions prevail, e.g., *Eurhynchium striatum* (L 5; F 5), *Isothecium alopecuroides* (L 5; F 5), *Polytrichum commune* (L 6; F 7) (Table 1). PERMANOVA test pointed to significant statistical differences between the occurrence of species on individual plantations (F = 7.426, p < 0.01).

**Figure 5:**
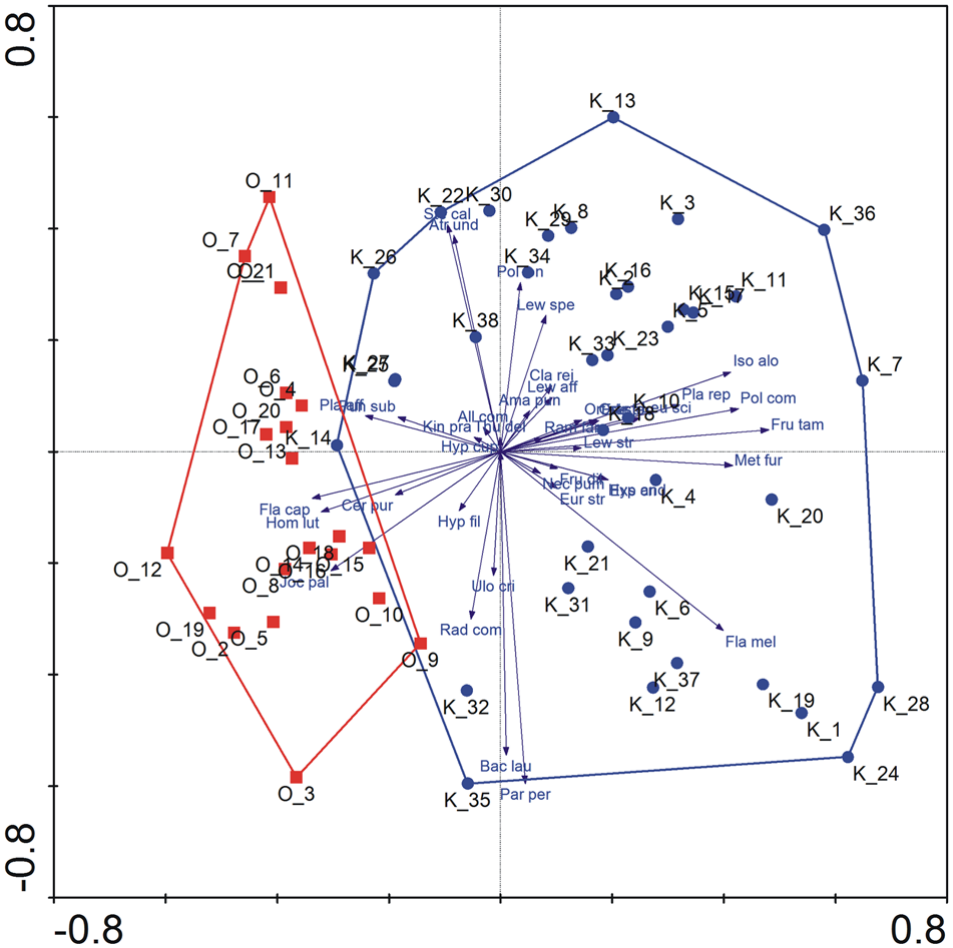
PCA of the tea bushes of the two studied plantations. Blue circles—bushes of the Kobuleti plantation, red squares—bushes of the Ozurgeti plantation. Marking numbers adequate to the numbering in Supplementary Table S2-S3.

The principal component analysis (PCA) also shows distinct difference between the researched plantations. This analysis showed the division of the studied tea bushes into two distinct groups, one group includes the bushes of the Ozurgeti plantation, the other Kobuleti (Fig. 5). Both main axes of PCA explain 21% of the variability in total. A clear division of the studied bushes of the two analyzed plantations also shows grouping by the Ward's method with the Euclidean measure (dendrogram) (Supplementary Fig. S1).The analysis of the species composition of the studied areas shows that 51% of species distinguish the studied plantations (Table 1; Supplementary Tables S2 and S3). The Shannon H index shows that the Kobuleti plantation (H = 3.343) is more diverse than the Ozurgeti—thus, there are more dominant species in Ozurgeti plantation (H = 2.960). Also, the permutation test (p < 0.001) indicates that the diversity indices for Kobuleti and Ozurgeti are statistically significantly different (Supplementary Fig. S2).

In the studied area (both plantations), lichens and mosses were recorded on three types of substrate: soil, bark and wood of tea shrubs; however, most taxa were found to grow on only a single substrate type (n = 32). Only one species (*Stereodon callichrous*) grew on all four (Table 1).

Typical forest taxa were found to dominate, e.g., *Eurhynchium striatum*, *Kindbergia praelonga*, *Plagiomnium affine*. Species with a broad ecological amplitude were recorded much less often (e.g., *Hypnum cupressiforme*, *Ceratodon purpureus* (Table 1). Among the recorded cryptograms, far more epiphytic taxa, such as *Frullania dilatata*, *Lewinskya speciosa* and *Punctelia subrudecta* (29 species), were observed than epixylic ones (five species), e.g., *Evernia prunastri*, *Hypnum cupressiforme* and *Jochenia pallescens* (Table 1).

Of the recorded cryptogams, some of the most interesting were epiphytic mosses and liverworts. This group includes bryophytes that are rare or even critically endangered in most European countries, e.g. *Orthotrichum stellatum* has been recorded there repeatedly and its populations appear richly fertile. Other rare species found in tea plantations include *Lewinskya striata*, *Orthotrichum* *stramineum*, *Hypnum andoi*, and *Stereodon callichrous* (Table 1). The recorded lichens species are widespread, albeit sometimes locally rare, in the Northern Hemisphere.

### Vertical distribution patterns and response of cryptogames to environmental factors

A fairly similar number of species occurred on the soil and in the lower part of the tea bushes of both plantations. On the soil in the area of the Ozurgeti and Kobuleti plantations, 12 and 14 taxa were recorded respectively (Table 1), while in the lower part of the shrubs of both plantations, three species were recorded. More pronounced differences in the number of species were noted in the middle and upper parts of the tea bushes of Ozurgeti and Kobuleti. In the middle part tea bushes of the Ozurgeti and Kobuleti 16 and 25 taxa were recorded, while in the upper part 11 and 20 species were recorded respectively (Table 1).

The analysis of the similarity of individual zones of tea shrubs (Jaccard's measure) showed that the upper and middle parts were the most different between the two plantations (upper d = 0.381; middle d = 0.429), while the lower parts of the shrubs were the most similar to each other (Supplementary Table S4). A comparison of the analyzed zones with each other of individual plantations in terms of the cryptogams recorded on their area (PERMANOVA, F = 6.154, p < 0.001) shows that the differences are statistically significant (Supplementary Table S5).

In terms of indicator numbers (Wirth^50^; Ellenberg et al.^51^) exclusive species of individual parts tea bushes indicated that the upper parts are inhabited by light-demanding species with medium moisture requirements, e.g. *Radula complanata* L = 7; F = 5). The middle part is covered with shade-loving with higher humidity requirements taxa, e.g., *Alleniella complanata* L = 4; F = 4, *Exsertotheca crispa* L = 4; F = 6, *Isothecium alopecuroides* L = 5; F = 5, *Platygyrium repens* L = 6; F = 4. On the other hand, species with a wide ecological amplitude in relation to the analyzed factors were recorded on the soil, e.g., *Plagiomnium affine*, *Eurhynchium striatum* L = 5; F = 5, *Thuidium delicatulum* L = 7; F = 4. In addition, the species exclusive to the Kobuleti plantation were found to have higher F factor values than those exclusive to the Ozurgeti plantations (Table 1). The species exclusive to the Ozurgeti plantation are characterised by much higher values of factor L than the other groups. However, the species common to both plantations have higher humidity factor values (F) than those found only on individual plantations.

**Figure 6:**
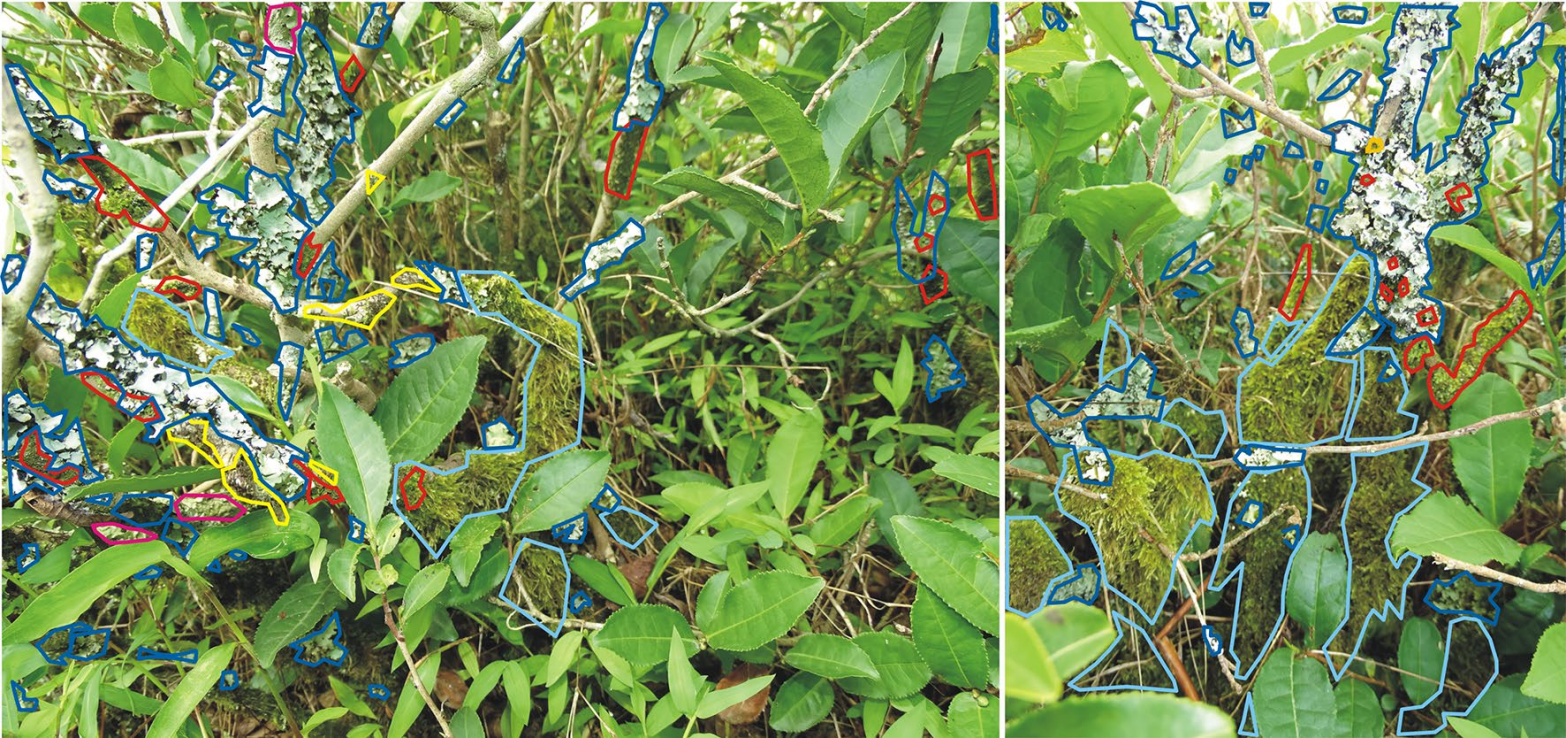
Distribution patterns of lichens and bryophytes of the Kobuleti plantation (photos by R. Piwowarczyk, 29 July 2018). Explanation: *Flavoparmelia caperata*—dark pink, *Hypnum cupressiforme*—blue, *Metzgeria furcate*—yellow, *Orthotrichum* sp.—orange, *Parmotrema perlatum*—dark blue, *Radula complanata*—red.

On the bushes of the Kobuleti tea plantation, the most common lichen is *Parmotrema perlatum*, with *Flavoparmelia caperata* being less frequently observed. The former also dominates in terms of occupied space. Both species were located in the middle and top parts of tea bushes (Fig. 6). Other lichens were recorded less frequently. Among the bryophytes, the most common are *Hypnum cupressiforme* and *Radula complanata*, with the former also dominating in terms of occupied area (Fig. 6). Other taxa growing on tea shrubs are much rarer or even sporadic. *Polytrichum longisetum*, *Atrichum undulatum* and *Stereodon callichrous* were observed on the soil around the tea bushes (Fig. 6); however, their presence was very sporadic.

Three cryptogam species dominate on the tea bushes in the Kobuleti plantation: *Hypnum cupressiforme* (occupying the lowest and middle parts of the bush), *Parmotrema perlatum* (middle and upper), and *Radula complanata* (upper parts of the tea bush). Other rarer and sporadic taxa can also be observed at the middle and top of the bushes (Fig. 6).

**Figure 7:**
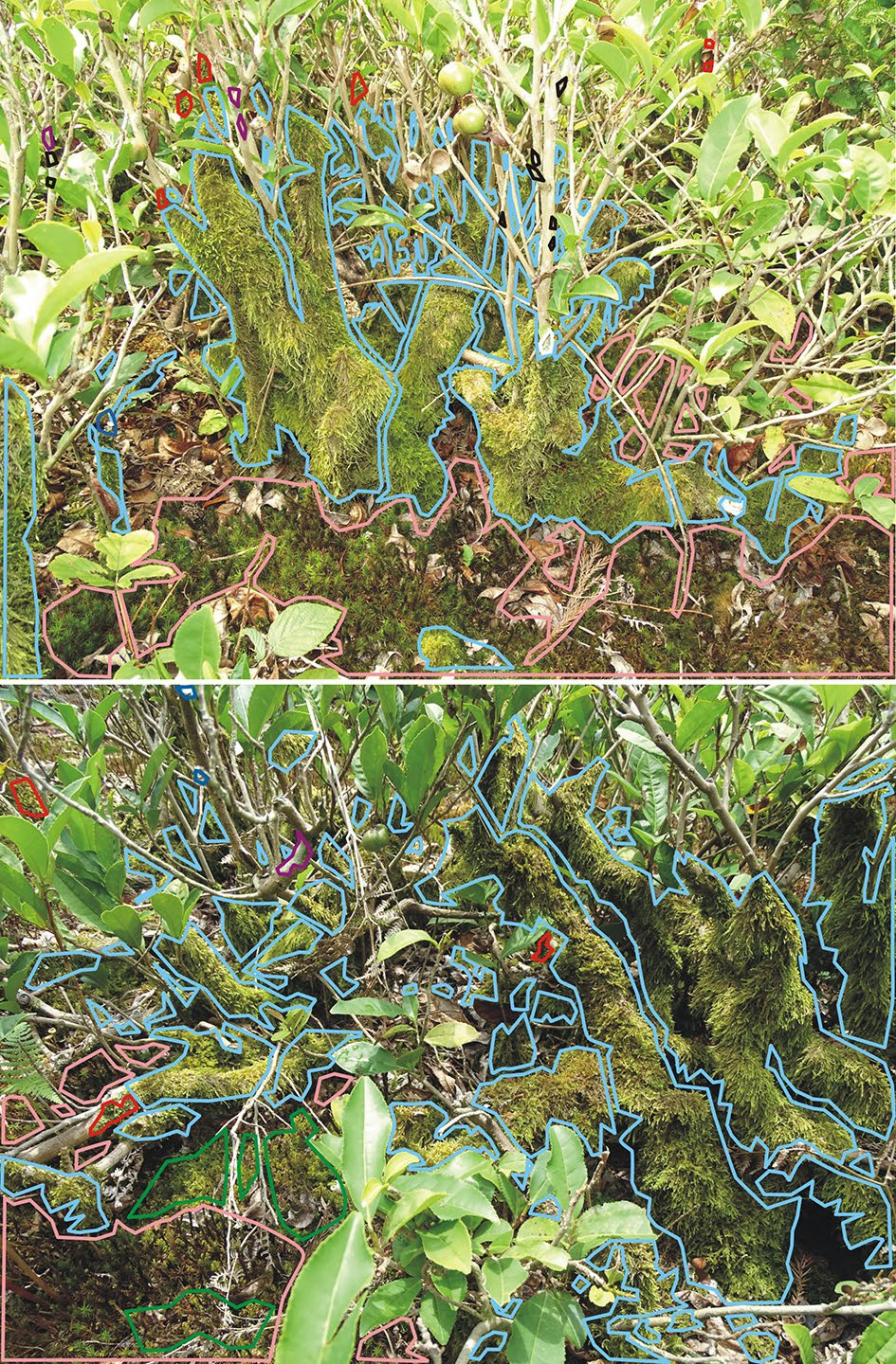
Distribution patterns of lichens and mosses—Ozurgeti plantation (photos by R. Piwowarczyk, 29 July 2018). Explanation: *Bacidia laurocerasi*—black, *Fellhanera*—dark purple, *Hypnum cupressiforme*—blue, *Parmotrema perlatum*—dark blue, *Plagomnium affine*—dark green, *Polytrichum longisetum*—pink, *Punctelia subrudecta*—white, *Radula complanata*—red.

The mosses clearly dominate on Ozurgeti tea bushes. Among the lichens, the most common are *Bacidia laurocerasi* and *Fellhanera* sp.; however, they take up a low percentage of the area compared to the bryophytes. Both lichen taxa tend to be located at the top of the tea bushes (Fig. 7). Other lichen species were recorded much less frequently. Among bryophytes, the most common is *Hypnum* *cupressiforme*, which also clearly dominates in terms of occupied space. Other taxa (e.g. *Radula complanata*) are rarer and were noted only in the top parts of the bushes. However, typical forest species were commonly found on the soil (Fig. 7).

The observed distribution patterns of Ozurgeti plantation cryptogams indicates that one species dominates on the shrubs of this plantation—*Hypnum cupressiforme*, occupying the lowest and middle parts of the bush, with the other parts being inhabited by rarer taxa such as *Bacidia laurocerasi*, *Fellhanera* sp. and *Radula complanata*. In addition, the ground around the bushes is covered by a dense turf of typical epigeic forest bryophytes, such as *Polytrichum* sp., *Atrichum undulatum*, *Plagiomnium affine* and *Stereodon callichrous* (Fig. 7).

The Shannon H index calculated for individual zones of both plantations indicates that the greatest diversity of cryptogams is characteristic of the middle and upper parts of the analyzed shrubs. The highest index was recorded for the Kobuletti plantation (medium 3.005; upper 2.800), but, on the other hand, the lowest biodiversity of the studied shrubs is in the low zones of both plantations (Supplementary Table S6). Additionally, the t-test indicates that the obtained differences between Shannon H indexes are statistically significant (p < 0.001) (Supplementary Table S7).

## Discussion

Tea plantations in terms of mosses, liverworts and lichens have never been studied in detail, in addition, these organisms have never been the main subject of analysis^54–57^. During the research, 39 taxa of cryptogams were recorded; this result is a fairly similar to the Gradstein et al.^57^ research and much higher than the research conducted by Tan et al.^56^. However, compared to the artcles cited above, conducted research are a much more detailed ecological analysis of ecological preferences of the described organisms.

Epiphytic bryophytes occur most often in forest vegetation or grow on the bark of solitary trees. Although their occurrence on tea plantations has not been studied in detail, it seems that such ecosystems can act as hot spots of rare and interesting bryophytes in Georgia.

The Kobuleti and Ozurgeti plantations represented similar differences between values of average minimum and maximum annual temperatures; however, Kobuleti was characterised by a higher average monthly minimum temperature than Ozurgeti, except for three months: March, April and May. Similarly, Kobuleti demonstrated higher average monthly maximum temperatures, except during March, April, May and June. Kobuleti is characterised by higher average annual precipitation (from 163.9 to 221.8 mm) than Ozurgeti (from 155.9 to 209.2 mm); these differences could be significant as the Kobuleti plantation is located in the lowlands, i.e. closer to the sea. In addition, Kobuleti also demonstrated higher average monthly precipitation in all months except April (in 2014, 2016 and 2018), June (from 2013 to 2017) and July (in 2013, 2015 and 2017)^49^.

Such differences in weather data influence the distribution and diversity of the analysed mosses and lichens; these can also be influenced by the management method of the tea plantations, as confirmed for other deciduous tree crops^58^. Hence, the Kobuleti plantation has greater species variety than Ozurgeti (Table 1), including more liverworts and typical epiphytes (e.g., *Frullania tamarisci*, *Metzgeria furcata*, *Lewinskya affinis*, *L*. *speciosa*, *Leucodon sciuroides*, *Neckera pumila*), but fewer species with a broad ecological spectrum, such as *Ceratodon purpureus*.

Additionally, our research has managed to show that the species composition of cryptogams in individual plantations depends on their management, state of preservation and thus on the habitat conditions prevailing there. This also influenced the qualitative and quantitative vertical distribution of analyzed organisms of individual tea shrubs. Our findings confirm that mosses, liverworts and lichens are effective bioindicators, both the habitat conditions in the studied phytocenosis and the overgrown substrates. This is confirmed by the well-known, but sometimes underestimated, great value in ecological research in plant communities^10–15,59–65^; they can even be used to identify heavy metal contamination in the environment based on their tissue concentrations^48^. The other recently published results also provide a comprehensive evaluation of epiphytic bryophytes as bio-indicators and also an estimated critical load for their survival in forest ecosytem^66^. It should be respected for active biodiversity conservation. Epiphytic bryophytes demonstrate clear vertical distribution patterns in tropical and temperate forests^67,68^. Cornelissen and Steege^69^ found the distribution of epiphytic species and their life-forms to be influenced by the vertical zones of host trees, and Lyons et al.^68^ report that bryophytes were more abundant in the lower and middle zones of trees. It has been proposed that these vertical distribution patterns can be explained by the wide microhabitat heterogeneity present throughout the vertical profile of host trees^70^ and the ability of epiphytes to colonize each microhabitat, according to their physiological requirements and adaptations^71^.

Our research also shows that lichen and bryophytes may respond differently to the loss of the typical habitats which, in this case, are forest ecosystems. Lichens seem to be more sensitive as few and common in many areas species were recored in studied tea plantations, in opposite to bryophytes, which include more rare and typical forest epithytes. Similar pattern was found by Czerepko et al.^72^ who showed that lichen species richness was significantly correlated with the degree of forest naturalness (with the highest number of species recorded in natural forests and lowest in managed forests), but bryophytes did not clearly responded to the management regimes. Also Putna and Mežaka^73^ found that bryophytes may not immediately respond to anthropogenic disturbance. This may be related to the low dispersal range of lichens or their specific habitat requirements, meanwhile bryophytes may be more plastic in adapting to different niches than lichens and can occur in sub-optimal habitats^72^.

Most epigeic or multi-substrate species are common or very common throughout Eurasia (e.g., *Atrichum undulatum*, *Ceratodon purpureus*, *Eurhynchium striatum*, *Hypnum cupressiforme*, *Jochenia pallescens*, *Kindbergia praelonga*, *Plagiomnium affine*, *Polytrichum longisetum*, *P*. *commune*, *Thuidium delicatulum*)^74–84^. In the nineteenth century, they were already noted by Brotherus^85^ in the Caucasus, and nowadays are considered common or very common in Georgia^86^. Other taxa, such as *Hypnum andoi* and *Stereodon callichrous*, are not so common^70–80,83^ and in Georgia they are considered rare^86,87^.

The recorded lichens species are widespread, albeit sometimes locally rare in the Northern Hemisphere (*Evernia prunastri*, *Ramalina farinacea*) or subcosmopolitan to cosmopolitan (*Amandinea punctata*, *Bacidia laurocerasi*, *Cladonia rei*, *Flavoparmelia caperata*, *Parmotrema perlatum*, *Punctelia subrudecta*)^52,88–91^. Most are typical epiphytic lichens, but sometimes they can grow on other substrates, e.g., wood or rocks^52,92^. Only *C. rei* belongs to a group of typically terricolous lichens^52,90^.

A total of 19 orthotrichaceous moss taxa, including *Lewinskya*, *Nyholmiella*, *Orthotrichum*, and *Pulvigera* according to Plášek et al.^93^; Lara et al.^94^; Sawicki et al.^95^ have so far been reported from Georgia^86,96–99^. During our study, five of them were recorded growing epiphytically in the studied tea plantation.

The family Orthotrichaceae is represented in the studied area by rare species, one of which being *Orthotrichum stellatum*, considered as very rare in many European countries and only recently found in Georgia. The species was collected in Georgia for the first time during a previous Polish botanical expedition in 2016, where it was collected from the bark of *Pterocarya fraxinifolia* Spach near a public road towards the Mitrala National Park^98^. To date, only five localities are known in the country, including the one in the present article^98,100^. Our present record is the first one of this species from tea plantations. This species is considered endangered or even critically endangered in most European countries^84^, but it was repeatedly recorded in our plots and its populations appear to be richly fertile. *Orthotrichum* *stellatum* is a species with a disjunct transatlantic distribution. It occurs in eastern North America, Europe and locally in western Asia. In Europe, its geographical range stretches from Norway, across Central Europe (Czech Republic, Hungary) to the Mediterranean and south-eastern Europe, extending to the Pontic Mountains in Turkey^101,102^.

Three other interesting species are *Lewinskya striata*, *L*. *affinis* and *Orthotrichum stramineum*, which have only a local distribution in Georgia. *Lewinskya* *striata* is a widespread species and common throughout Europe, but also occurs rather sparsely in north Africa, southern and eastern Asia, China and North America^45,103–105^. *Lewinskya affinis* was reported as ‘common’ in Georgia by Chikovani and Svanidze^86^ and by Eckstein and Zündorf^100^, but only in mountain regions. Therefore, its rich occurrence in the study areas in the lower parts of the country is surprising. Similarly, while *O*. *stramineum* has been reported only from the northern part of the country so far^100^, it was repeatedly observed in the southwestern part of the country in the present study. In addition, while *O*. *stramineum* was for long time considered to be a European species, it has been identified in North America^45,106^ and later in China^108^.

The vertical distribution of cryptogams of tea shrubs has not been the subject of research so far. In individual studies, their location on individual substrates was only indicated^104–107^. However, this vertical distribution appear very similar to those observed in forest communities, while the ground is covered by bryophytes with a wide ecological amplitude. However, obligatory epiphytes predominate in the higher zones, probably due to their predispositions that arose during their evolution: they do not typically become well established in the lower zones due to the higher competition from other species. Despite tea shurbs being considerably shorter, the same patterns of vertical distribution were demonstrated on the host shrubs in tea plantations and on trees in forests.

Due to the fact that detailed research of epiphytic bryophytes was performed only on the area of tea plantations, we do not have relevant data available for comparison with the species diversity of bryophytes growing in other communities in the area. However, the observations in other regions show that the diversity of mosses growing epiphytically on old solitary trees, terrestrially on open soil or on the surface of stones along plantations is significantly lower^6,19,108^. This is mainly due to influence of the microclimatic conditions. The bark of old trees is exposed to the significant effects of long-term drying out due to sunlight and the effects of wind. These conditions limit the number of species occuring in these habitats. Likewise, the microclimatic conditions are similar on stones and boulders along the plantations. They are avaible only for the species which have significant ecological adaptations. On the other hand, bare soil is under the influence of the succession which continuously decrease the diversity of bryophytes by overgrowth of vascular plants. So it can be concluded that thanks to the slight shading and higher humidity, the vegetation of the plantations offers conditions that suit epiphytic species and therefore the species richness of bryophytes in these communities is so high. These habitats can therefore be assessed as an important hotspot in the landscape, they provide long-term suitable conditions for the survival and development of epiphytic bryophyte and lichens communities and moreover operate as a center for the distribution of their spores to the surrounding environment. Therefore, it is necessary to pay attention to such ecosystems it terms of nature protection^33,46,61^.

## Conclusions

This article presents the species diversity and spatial arrangement of epiphytic bryophytes and lichens in two Georgian tea plantations, indicating the importance of such environments in providing host plants and hot spots for cryptogams. The study also examines the occurrence of these cryptograms in terms of their phytogeography, environmental conditions and ecological indicators. Our research indicates that of the 39 identified moss and lichen taxa, forest species such as *Frullania dilatata* and *Lewinskya speciosa* predominate. These species, as well as, among others: *Orthotrichum stellatum*, *O*. *stramineum*, *Lewinskya striata*, and *L*. *affinis* due to the fact that they are rare or sparse in Georgia, and additionally taking into account the fact that obligatory epiphytes are considered an outstandingly bioindication group of organizations, it can be concluded that this group of cryptogams is one of the most interesting and important elements of the studied plantations.

The studied tea plantations differ in terms of their habitat conditions, which is reflected in the bryophytes and lichens recorded there. However, despite some differences, the two tea plantations generally displayed similar species distribution patterns for mosses and lichens: species with a wide ecological amplitude and multi-substrate preferences inhabited the lower parts of the shrubs, while those with a narrow ecological scale, i.e. epiphytes, occupied the highest zones. This division highlights the strong bioindication properties of both groups of organisms. Interestingly, a similar vertical distribution of species can be seen in all types of natural or semi-natural forests, particularly deciduous ones.

## Supplementary Information

Supplementary Information.

## Data Availability

We declare that all data on the basis of which this manuscript was created are publicly available and disseminated in the manuscript itself or as supplementary materials.
