# Supplementary material for: Tea plantations and their importance as host plants and hot spots for epiphytic cryptogams
Source: Sci Rep. 2021 Sep 14;11:18242. doi: 10.1038/s41598-021-97315-2 (PMC8440766; doi:10.1038/s41598-021-97315-2)
Supplement: Supplementary file 1 — Supplementary Information. [file 41598_2021_97315_MOESM1_ESM.pdf]

Tea plantations and their importance as host plants and hot spots for epiphytic cryptogams

Grzegorz J. Wolski[1*], Renata Piwowarczyk[2], Vítězslav Plášek[3], Martin Kukwa[4], Karolina Ruraż[2]

[1]University of Lodz, Faculty of Biology and Environmental Protection, Department of Geobotany and Plant Ecology, Banacha 12/16, PL-90-237 Lodz, Poland. E-mail: grzegorz.wolski@biol.uni.lodz.pl **corresponding author*

[2]Jan Kochanowski University, Institute of Biology, Department of Environmental Biology, Center for Research and Conservation of Biodiveristy, Uniwersytecka 7, PL-25-406 Kielce, Poland. E-mail: piwowarczyk@ujk.edu.pl; karolina.ruraz@ujk.edu.pl

[3]University of Ostrava, Deptartment of Biology and Ecology, Chittussiho 10, CZ-710 00 Ostrava, Czech Republic. E-mail: vitezslav.plasek@osu.cz

[4]University of Gdańsk, Faculty of Biology, Department of Plant Taxonomy and Nature Conservation, Wita Stwosza 59, PL-80-308 Gdańsk, Poland. E-mail: martin.kukwa@ug.edu.pl

Table S1. (1/2) Climatic information of the two localities on tea plantations.

	Minimum temperature (°C)											
	2013		2014		2015		2016		2017		2018	
	Kobuleti	Ozurgeti	Kobuleti	Ozurgeti	Kobuleti	Ozurgeti	Kobuleti	Ozurgeti	Kobuleti	Ozurgeti	Kobuleti	Ozurgeti
Jan	3.7	3.6	3.1	3.0	3.4	3.2	2.7	2.6	1.0	1.0	5.7	5.4
Feb	5.5	5.2	4.0	3.5	4.7	4.3	6.2	5.9	0.2	-0.1	6.7	6.3
Mar	5.5	5.8	6.0	6.2	3.8	4.2	5.4	5.8	4.6	5.0	7.6	7.8
Apr	7.6	8.2	7.2	7.7	6.2	6.7	7.7	8.2	5.8	6.4	7.8	8.2
May	13.1	13.3	11.9	12.3	10.8	11.1	11.6	11.8	11.0	11.2	12.7	13.0
Jun	16.9	16.9	16.0	16.0	16.9	16.9	16.6	16.5	16.2	16.1	16.9	16.9
Jul	18.7	18.6	19.3	19.3	19.7	19.6	19.1	19.0	19.8	19.6	20.6	20.5
Aug	19.1	18.7	21.2	20.9	20.1	19.7	21.1	20.8	20.9	20.7	-	-
Sep	15.8	15.4	17.1	16.8	18.3	18.0	15.9	15.5	18.2	17.8	-	-
Oct	9.7	9.5	13.1	12.6	13.2	12.9	11.2	10.9	11.4	11.2	-	-
Nov	7.9	7.5	7.8	7.4	7.2	6.9	5.7	5.3	7.8	7.3	-	-

Dec	-0.4	-0.7	7.2	6.8	2.7	2.5	0.9	0.5	5.6	5.3	-	-
Annual	10.3	10.2	11.2	11.0	10.6	10.5	10.3	10.2	10.2	10.1	-	-
	Maximum temperature (°C)											
Jan	11.5	11.2	11.4	11.0	11.3	11.0	10.1	9.9	8.8	8.6	12.6	12.1
Feb	13.7	13.4	14.1	13.5	13.6	13.3	14.7	14.4	9.9	9.6	15.4	15.0
Mar	15.5	15.9	17.2	17.5	13.5	13.9	15.8	16.3	15.4	15.9	17.8	18.1
Apr	19.0	19.5	19.3	19.8	17.0	17.6	20.5	21.0	17.8	18.3	19.5	20.0
May	21.3	21.8	21.9	22.4	20.8	21.2	21.2	21.6	20.9	21.2	21.6	22.0
Jun	26.0	26.3	25.8	26.0	25.3	25.6	25.0	25.2	25.7	25.9	26.0	26.3
Jul	26.1	26.0	27.4	27.4	27.4	27.4	26.5	26.5	28.2	28.2	28.4	28.5
Aug	26.7	26.6	29.1	29.0	27.8	27.7	29.2	29.1	29.3	29.3	-	-
Sep	23.9	23.7	24.8	24.7	27.1	27.1	23.4	23.4	27.1	27.0	-	-
Oct	19.6	19.5	21.3	20.9	20.6	20.4	20.6	20.3	20.8	20.7	-	-
Nov	17.1	16.7	15.7	15.4	17.4	17.1	15.6	15.1	17.0	16.6	-	-
Dec	8.4	8.2	13.8	13.4	11.1	10.9	8.6	8.3	14.1	13.9	-	-
Annual	19.1	19.1	20.2	20.1	19.4	19.4	19.3	19.3	19.6	19.6	-	-

Table S1. (2/2) (continued)

	Precipitation (mm)											
	2013		2014		2015		2016		2017		2018	
	Kobuleti	Ozurgeti	Kobuleti	Ozurgeti	Kobuleti	Ozurgeti	Kobuleti	Ozurgeti	Kobuleti	Ozurgeti	Kobuleti	Ozurgeti
Jan	242.6	215.5	236.8	222.1	178.3	158.2	327.0	310.6	178.5	168.6	247.5	235.6
Feb	167.1	144.4	81.9	78.6	169.2	151.0	104.1	99.9	115.1	108.8	145.3	136.3
Mar	208.6	199.7	147.1	139.8	152.8	142.1	186.0	174.7	108.4	107.3	237.4	225.8
Apr	88.6	85.9	77.3	80.0	158.6	155.2	109.5	109.7	94.2	91.8	43.3	46.6
May	72.7	70.5	82.7	80.1	75.6	73.0	115.0	106.8	130.2	126.9	104.3	95.9
Jun	143.0	150.4	137.6	145.1	148.6	149.9	218.6	226.3	131.9	143.9	145.5	142.1
Jul	163.1	168.3	156.2	154.2	63.4	65.1	198.5	193.9	90.8	92.6	153.5	150.8
Aug	138.1	130.0	82.4	80.7	259.8	231.1	212.3	190.2	187.9	178.3	-	-
Sep	351.8	339.0	434.2	417.2	93.6	86.3	448.8	417.2	141.7	138.8	-	-
Oct	232.3	214.3	197.4	172.8	369.0	334.4	222.6	205.6	359.5	319.7	-	-
Nov	128.7	118.0	278.8	248.8	298.5	272.8	164.9	147.5	184.5	165.8	-	-
Dec	209.6	203.1	176.6	168.4	191.6	189.7	354.6	328.1	244.5	228.0	-	-
Annual	178.8	169.9	174.1	165.7	179.9	167.4	221.8	209.2	163.9	155.9	-	-

Table. S2. Presence of individual species (and its six-letter code) on the studied tea bushes in Ozurgeti plantation.

**Species**	**Code**	**O1**	**O2**	**O3**	**O4**	**O5**	**O6**	**O 7**	**O 8**	**O 9**	**O 10**	**O 11**	**O 12**	**O 13**	**O 14**	**O 15**	**O 16**	**O 17**	**O 18**	**O 19**	**O 20**	**O 21**
Frullania dilatata	*Fru dil*				1		1		1	1	1			1	1	1			1	1		1
Radula complanata	*Rad com*	1	1	1		1	1		1	1	1		1	1	1		1	1	1	1	1	1
Alleniella complanata	*All com*					1		1				1			1		1			1	1	1
Atrichum undulatum	*Atr und*	1			1			1	1			1		1	1		1				1	
Ceratodon purpureus	*Cer pur*				1				1											1		
Exsertotheca crispa	*Exs cri*				1			1	1			1			1		1	1	1		1	
Hypnum andoi	*Hyp and*			1		1	1			1	1			1		1	1		1		1	1
H. cupressiforme	*Hyp cup*	1	1	1	1	1	1	1	1	1	1	1	1	1	1	1	1	1	1	1	1	1
H. cupressiforme var. *filiforme*	*Hyp fil*	1	1	1		1	1	1	1		1	1	1	1	1	1	1	1		1	1	1
Homalothecium lutescens	*Hom lut*		1	1	1		1		1			1		1		1				1		
Jochenia pallescens	*Joc pal*			1		1			1				1					1	1	1		
Kindbergia praelonga	*Kin pra*		1												1							
Lewinskya striata	*Lew str*				1					1												
Orthotrichum stellatum	*Ort ste*	1																				
Polytrichum longisetum	*Pol lon*						1					1		1		1					1	
Plagiomnium affine	*Pla aff*	1				1	1	1				1	1	1						1	1	1
Stereodon callichrous	*Ste cal*	1			1	1	1	1		1		1	1		1		1	1	1		1	1
Thuidium delicatulum	*Thu del*							1														
Ulota crispa	*Ulo cri*		1	1			1			1			1			1					1	1
Bacidia laurocerasi	*Bac lau*		1	1		1				1			1	1	1		1		1			
Evernia prunastri	*Eve pru*										1	1										1
Flavoparmelia caperata	*Fla cap*		1		1			1			1		1		1		1	1	1		1	1
Parmotrema perlatum	*Par per*			1	1	1			1	1	1				1	1	1			1	1	

Species	Code	O1	O2	O3	O4	O5	O6	O 7	O 8	O 9	O 10	O 11	O 12	O 13	O 14	O 15	O 16	O 17	O 18	O 19	O 20	O 21
Punctelia subrudecta	*Pun sub*											1	1									

Table. S3. Presence of individual species (and its six-letter code) on the studied tea bushes in Kobuleti plantation.

Species	Code	K 1	K 2	K 3	K 4	K 5	K 6	K 7	K 8	K 9	K 10	K 11	K 12	K 13	K 14	K 15	K 16	K 17	K 18	K 19	K 20	K 21	K 22	K 23	K 24	K 25	K 26	K 27	K 28	K 29	K 30	K 31	K 32	K 33	K 34	K 35	K 36	K 37	K 38
Frullania dilatata	*Fru dil*	1	1		1	1			1			1	1	1			1		1			1		1					1			1			1		1	1	1
F. tamarisci	*Fru tam*	1	1	1		1		1	1			1	1				1	1		1	1			1	1				1	1		1					1	1	1
Metzgeria furcata	*Met fur*	1		1				1			1	1					1				1				1				1			1			1		1	1	
Radula complanata	*Rad com*	1	1		1		1	1		1	1	1			1	1		1	1	1	1		1		1	1	1		1	1		1	1	1	1	1		1	1
Alleniella complanata	*All com*	1	1		1	1	1				1	1			1		1	1	1				1			1	1	1				1	1					1	
Atrichum undulatum	*Atr und*		1	1		1			1		1			1		1				1			1				1			1					1		1		
Eurhynchium striatum	*Eur str*			1					1				1							1								1								1			
Exsertotheca crispa	*Exs cri*	1	1	1		1	1	1	1		1		1		1		1		1	1	1			1	1				1			1		1		1	1		
Hypnum andoi	*Hyp and*	1		1	1		1	1	1	1		1		1		1		1		1	1	1			1	1				1					1		1		
H. cupressiforme	*Hyp cup*	1	1	1	1	1	1	1	1	1	1	1	1	1	1	1	1	1	1	1	1	1	1	1	1	1	1	1	1	1	1	1	1	1	1	1	1	1	1
H. cupressiforme var. *filiforme*	*Hyp fil*	1	1	1		1	1		1	1	1	1	1			1		1		1	1		1	1	1		1	1	1		1	1		1	1	1		1	
Isothecium alopecuroides	*Iso alo*	1	1		1	1	1	1		1		1		1		1		1	1	1			1	1			1		1		1			1			1	1	
Kindbergia praelonga	*Kin pra*								1							1																							
Lewinskya affinis	*Lew aff*			1			1					1			1							1									1								
L. speciosa	*Lew spe*			1	1									1														1		1					1				
L. striata	*Lew str*		1									1				1				1	1		1		1			1			1			1		1			
Leucodon sciuroides	*Leu sci*		1					1		1			1			1		1	1				1						1						1				
Neckera pumila	*Nec pum*																													1			1			1	1		
Orthotrichum stellatum	*Ort ste*		1		1						1		1			1					1			1					1					1					
O. stramineum	*Ort str*			1					1								1												1						1	1			
Polytrichum commune	*Pol com*			1	1		1	1	1	1	1	1		1		1		1	1	1	1	1			1	1			1		1		1				1		1
P. longisetum	*Pol lon*					1						1		1			1				1		1								1			1			1		

Species	Code	K 1	K 2	K 3	K 4	K 5	K 6	K 7	K 8	K 9	K 10	K 11	K 12	K 13	K 14	K 15	K 16	K 17	K 18	K 19	K 20	K 21	K 22	K 23	K 24	K 25	K 26	K 27	K 28	K 29	K 30	K 31	K 32	K 33	K 34	K 35	K 36	K 37	K 38
Plagiomnium affine	*Pla aff*				1		1		1			1			1							1		1		1		1			1	1	1			1			1
Platygyrium repens	*Pla rep*	1			1	1	1					1		1		1	1	1				1		1	1					1		1		1					
Stereodon callichrous	*Ste cal*		1	1		1	1	1	1	1		1		1	1		1	1			1	1	1			1	1	1		1	1				1		1	1	1
Thuidium delicatulum	*Thu del*						1												1																				
Ulota crispa	*Ulo cri*		1		1							1					1		1	1					1	1		1				1				1		1	
Amandinea punctata	*Ama pun*								1										1			1						1		1		1							
Bacidia laurocerasi	*Bac lau*	1				1	1			1			1		1					1		1			1				1				1			1		1	
Cladonia rei	*Cla rei*		1		1				1																														
Evernia prunastri	*Eve pru*				1			1			1					1									1					1				1	1			1	
Flavoparmelia caperata	*Fla mel*	1				1	1	1		1	1		1							1	1	1			1			1	1				1			1	1	1	
Parmotrema perlatum	*Par per*	1			1		1			1			1							1	1	1			1				1			1	1			1		1	
Ramalina farinacea	*Ram far*				1										1										1						1				1				

Table S4. Analysis of the similarity (Jaccard index) of individual zones of tea bushes in the studied plantations.

		Ozurgeti				Kobuleti			
		soil	low	medium	upper	soil	low	medium	upper
Ozurgeti	soil	1.000	0.250	0.286	0.048	0.625	0.250	0.156	0.033
	low	0.250	1.000	0.200	0.000	0.214	1.000	0.120	0.000
	medium	0.286	0.200	1.000	0.389	0.208	0.200	0.429	0.214
	upper	0.048	0.000	0.389	1.000	0.043	0.000	0.207	0.381

Table S5. The PERMANOVA for the individual plantations and analyzed zones of tea bushes.

ALL	
Permutation N:	9999
Total sum of squares:	14.78

Within-group sum of squares:	13.08
F:	7.426
p:	0.0001
SOIL	
Permutation N:	9999
Total sum of squares:	11.46
Within-group sum of squares:	10.64
F:	4.384
p:	0.0001
LOW	
Permutation N:	9999
Total sum of squares:	4.065
Within-group sum of squares:	3.983
F:	1.179
p:	0.338
MEDIUM	
Permutation N:	9999
Total sum of squares:	14.4
Within-group sum of squares:	12.66
F:	7.812
p:	0.0001
UPPER	

Permutation N:	9999
Total sum of squares:	18.75
Within-group sum of squares:	16.8
F:	6.615
p:	0.0001

Table S6. The Shannon H index of individual zones of teh tea bushes in the studied plantations.

		soil	-95% CI	+95% CI	lower	-95% CI	+95% CI	medium	-95% CI	+95% CI	upper	-95% CI	+95% CI
OZURGETI	Number of species	12	12	12	3	3	3	15	15	15	10	10	10
	Shannon_H	2.224	2.115	2.333	1.085	1.031	1.140	2.501	2.415	2.588	2.054	1.942	2.166
KOBULETI	Number of species	14	14	14	3	3	3	25	25	25	19	19	19
	Shannon_H	2.358	2.270	2.447	1.077	1.030	1.124	3.055	2.998	3.113	2.800	2.727	2.872

Table S7. T test for the Shannon index of individual zones of the studied plantations.

	Ozurgeti	Kobuleti	t	p
Soil	2.224	2.358	-1.649	0.101
Low	1.085	1.0772	0.209	0.835
Medium	2.501	3.055	-9.36	<0.001
Upper	2.054	2.8	-9.39	<0.001

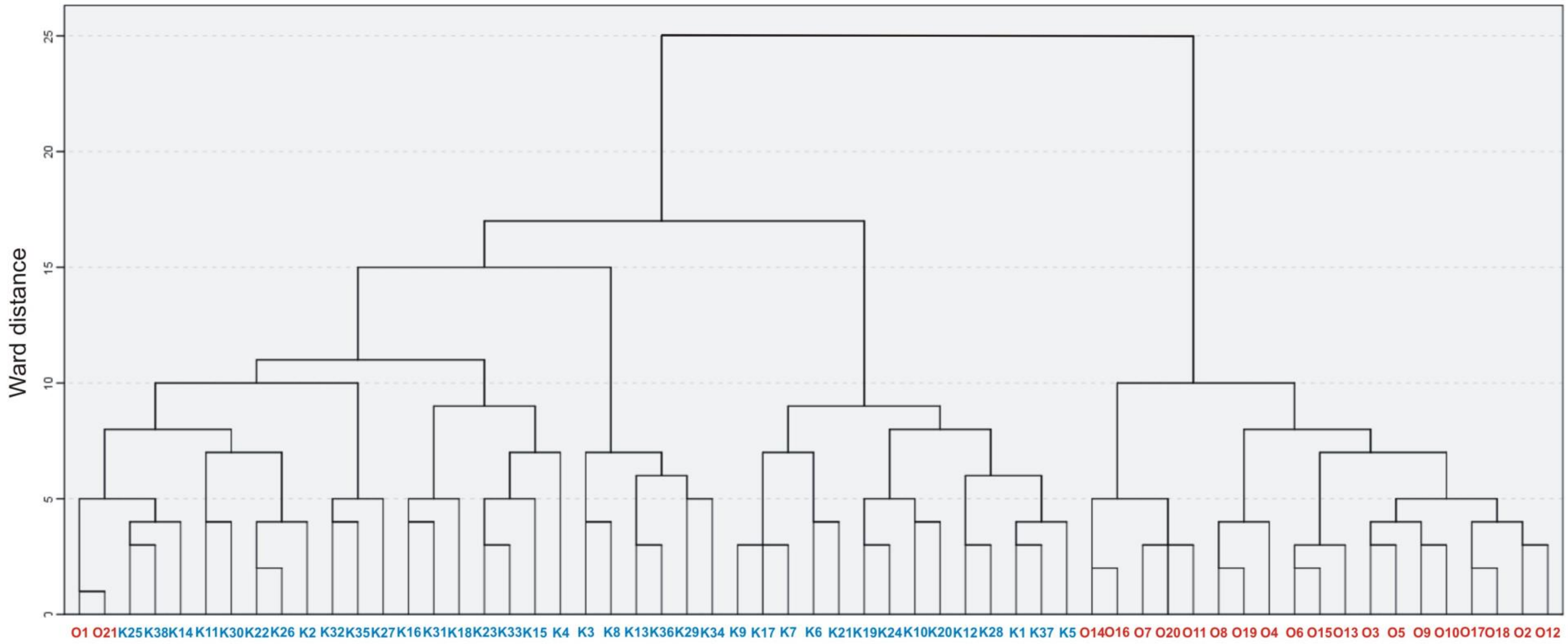

Fig. S1. Dendrogram of analyzed tea bushes. The blue markings are for Kobuleti, the red ones are for the Ozurgeti plantation; the numbers correspond to the numbering in tables S2 and S3.

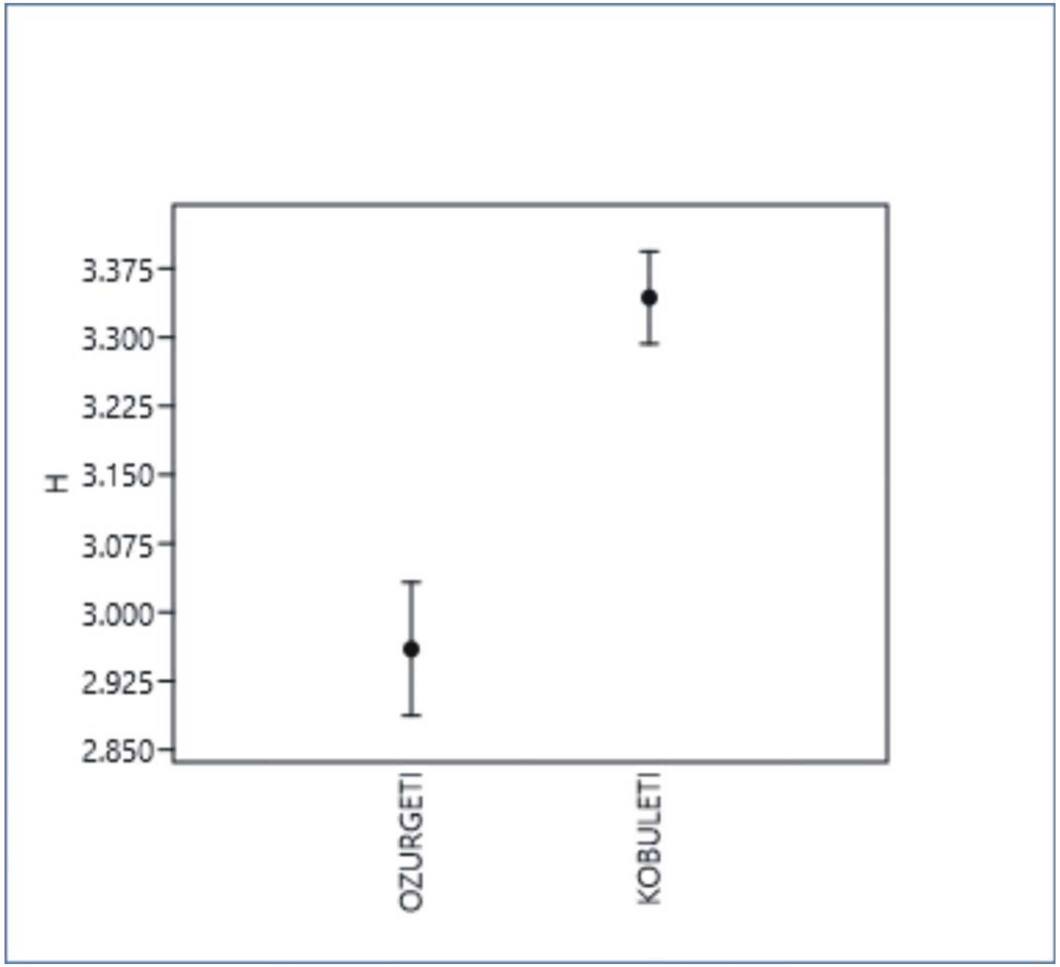

Fig. S2. The Shannon H index for Ozurgeti and Kobuleti plantation.
